# Bilateral Lung Consolidation with Static and Dynamic Air Bronchograms on Transesophageal Echocardiography

**DOI:** 10.3390/diagnostics16193224

**Published:** 2026-10-06

**Authors:** Aibek E. Mirrakhimov

**Affiliations:** Department of Anesthesiology, Perioperative, Critical Care, and Pain Medicine, Division of Adult Cardiothoracic Anesthesiology, University of Kentucky, Lexington, KY 40536, USA; ami276@uky.edu

**Keywords:** transesophageal echocardiography, lung consolidation, pneumonia, air bronchograms

## Abstract

Transesophageal echocardiography (TEE) is performed primarily for cardiac indications, but the acoustic window frequently includes portions of the lung, particularly once normal aeration is lost. We present the intraoperative TEE findings in a 56-year-old woman with ischemic cardiomyopathy who underwent delayed sternal closure after exchange of a durable left ventricular assist device (LVAD) for pump thrombosis. In addition to the expected assessment of device position, flow profiles, interventricular septal position, and right ventricular systolic function, the examination incidentally demonstrated bilateral lung consolidation containing multiple punctate and branching hyperechoic structures. On qualitative assessment of the recorded clips, some structures appeared relatively stationary, whereas others showed apparent movement, suggestive of static and dynamic air bronchograms. Bronchogram displacement was not measured, and no synchronized respiratory tracing was available to confirm inspiratory timing. The coexistence of these appearances may have reflected heterogeneous regional pulmonary pathology and did not, by itself, establish a single diagnosis. The patient subsequently required reintubation, frequent pulmonary toileting, and tracheostomy. These images illustrate that pulmonary abnormalities may appear within the transesophageal window when TEE is otherwise indicated, while emphasizing the limitations of qualitative assessment of bronchogram motion.

**Figure 1:**
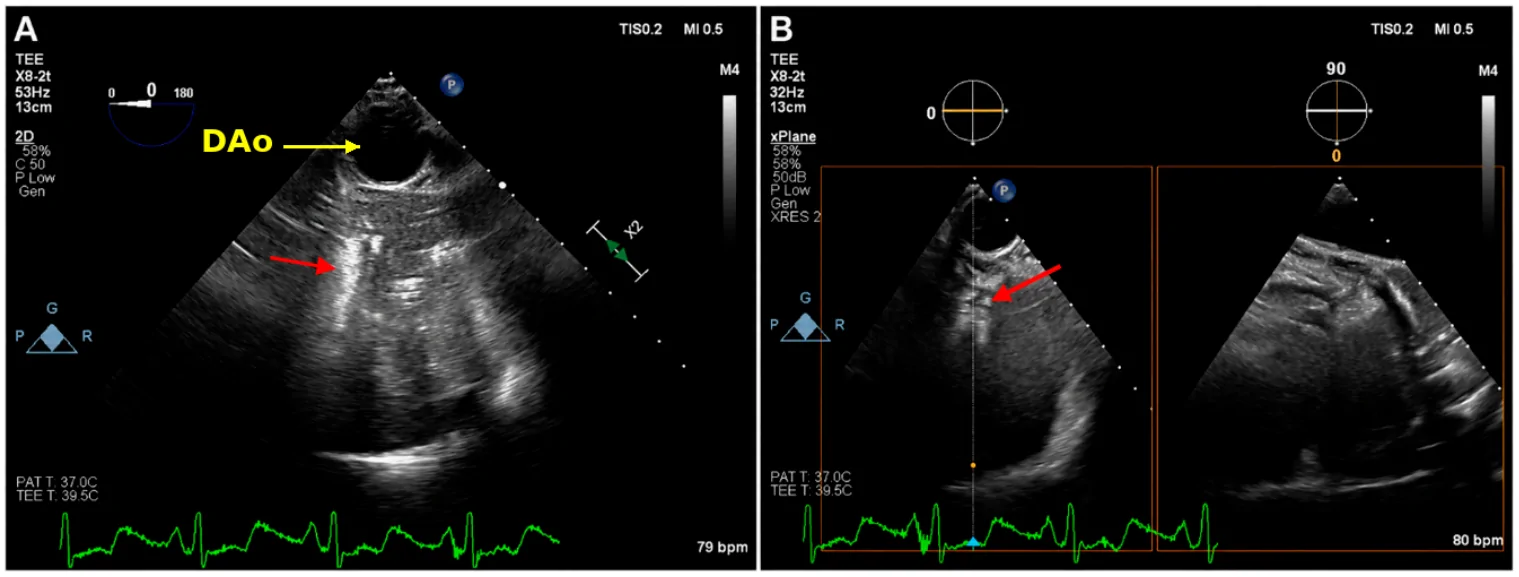
Intraoperative transesophageal echocardiography (TEE) demonstrating lung consolidation with air bronchograms (red arrows). (**A**) Left lung consolidation with air bronchograms. The descending thoracic aorta is labeled DAo (yellow arrow). A 56-year-old woman with ischemic cardiomyopathy supported by a durable left ventricular assist device (LVAD) presented with recurrent high-power alarms concerning for pump thrombosis. Her course after LVAD implantation had been complicated by an intra-abdominal abscess requiring chronic suppressive antibiotic therapy. She underwent urgent LVAD exchange with cardiopulmonary bypass, and thrombus was identified within the explanted pump. Because of persistent coagulopathy, the chest was left open. Two days later, she returned to the operating room for delayed sternal closure. Intraoperative TEE demonstrated appropriate position and flow profiles of the LVAD inflow cannula and outflow graft, stable interventricular septal position, and unchanged right ventricular systolic function. Examination beyond the cardiac structures incidentally demonstrated bilateral lung consolidation. TEE is not a standard pulmonary imaging modality; however, when otherwise indicated, it can visualize posterior and dependent lung regions once normal aeration is lost. Tsubo et al. demonstrated that TEE-detected dependent lung density correlated with computed tomography in patients with acute lung injury, and Verhaeghen et al. subsequently described systematic pulmonary assessment using TEE [1,2]. When alveolar gas is replaced by fluid, inflammatory material, blood, or collapse, the lung assumes an echotexture resembling the liver, and residual air within the bronchi appears as punctate or branching hyperechoic structures. On qualitative assessment of the available recording, some air bronchograms appeared relatively stationary, whereas others showed apparent movement, suggestive of static and dynamic air bronchograms (Video S1). Bronchogram displacement was not measured, and no synchronized respiratory or ventilator tracing was recorded; therefore, inspiratory timing could not be independently confirmed from the stored clips. The short duration of the left-sided recording further limited assessment across repeated respiratory cycles. (**B**) X-plane view of the left lung consolidation demonstrating air bronchograms.

**Figure 2:**
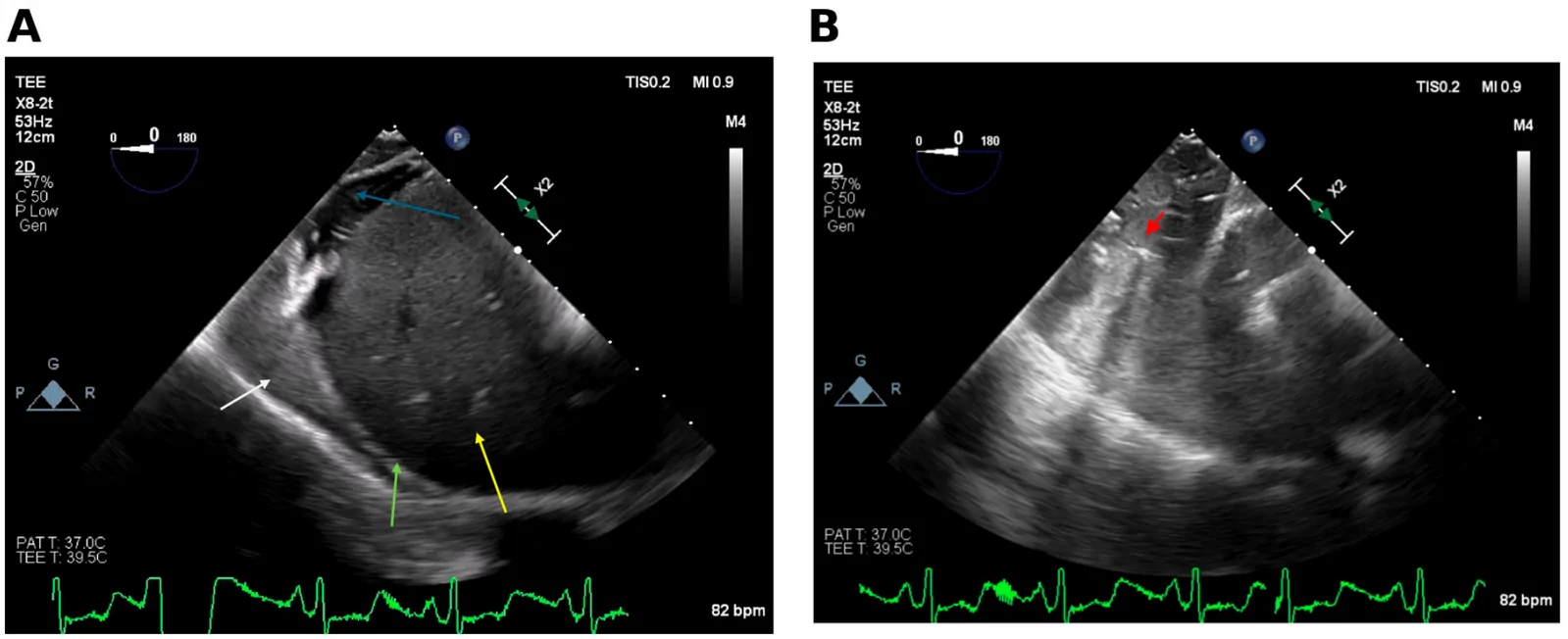
(**A**) TEE demonstrating the liver (yellow arrow), right hemidiaphragm (green arrow), perihepatic ascites (blue arrow), and right lung consolidation (white arrow). (**B**) Following withdrawal of the probe from the position shown in panel A, TEE demonstrates right lung consolidation with air bronchograms (red arrow). Lateralization of pulmonary findings during TEE is established by reference to adjacent anatomic landmarks rather than by image orientation alone. The descending thoracic aorta provides a reference for left-sided imaging, as labeled in Figure 1A. The liver and right hemidiaphragm provide anatomic orientation for the right-sided findings in Figure 2A. The right atrium and venae cavae may provide additional orientation when visible but are not shown in the selected panels.

A systematic approach to transesophageal lung ultrasonography, including probe rotation sequence and the assessment of regional aeration, has been described by Cavayas et al. [3]. Brault et al. subsequently described a bilateral protocol assessing upper and lower lung regions through sequential probe rotation toward the left and right shoulders [4], and Adi et al. reported clinical application in hypoxemic patients with COVID-19 [5]. More recently, Cao et al. described a semiquantitative transesophageal lung ultrasound score in mechanically ventilated critically ill patients, with grade 3 including consolidation with or without air bronchograms; their assessment was limited to the left hemithorax because of technical and anatomic constraints [6]. Our right-sided findings complement this literature and other intensive care applications [7], illustrating the feasibility of bilateral assessment in selected patients rather than establishing bilaterality as a new technique. Within the right-sided consolidation, some air bronchograms appeared relatively stationary, whereas others showed apparent movement on qualitative assessment (Video S2); these observations remain subject to the limitations described above. Static air bronchograms may occur in both atelectasis and pneumonia. By contrast, dynamic air bronchograms reflect inspiratory movement of air through patent bronchi and, on transthoracic lung ultrasonography, are highly specific but incompletely sensitive for pneumonia rather than resorptive atelectasis [8]. These transthoracic diagnostic estimates cannot be directly applied to the present qualitative transesophageal observations. In our patient, the differing appearances may have reflected heterogeneous regional pathology and did not establish a single diagnosis, and they preceded a prolonged respiratory course. The patient was extubated on postoperative day 2 after chest closure but soon required reintubation and frequent pulmonary toileting for thick secretions. A tracheostomy was subsequently placed. She was later decannulated and discharged home on hospital day 42. Lung consolidation and bronchogram behavior encountered within the transesophageal window may provide immediate complementary information but should be interpreted in conjunction with the clinical context and conventional pulmonary imaging.

## Data Availability

The original contributions presented in this study are included in the article. Further inquiries can be directed to the corresponding author.

## Supplementary Materials

The following supporting information can be downloaded at https://www.mdpi.com/article/10.3390/diagnostics16193224/s1: Video S1. TEE with X-plane imaging demonstrating left-sided lung consolidation with dynamic and static air bronchograms and air-filled bronchi corresponding to Figure 1; Video S2. TEE sweep demonstrating the liver with small-volume ascites and right-sided lung consolidation with both dynamic and static air bronchograms corresponding to Figure 2.
